# Incidence and Time to Return to Training for Stress Fractures during Military Basic Training

**DOI:** 10.1155/2014/282980

**Published:** 2014-01-21

**Authors:** Alexander M. Wood, Richard Hales, Andre Keenan, Alexandra Moss, Michael Chapman, Trish Davey, Andrew Nelstrop

**Affiliations:** ^1^Institute of Naval Medicine, Alverstoke, Hants PO12 2DL, UK; ^2^Commando Training Centre Royal Marines, Lympstone, Devon EX8 5AR, UK; ^3^Royal Army Medical Corps, Sandhurst GU15 4PQ, UK

## Abstract

Currently, little is known about the length of time required to rehabilitate patients from stress fractures and their return to preinjury level of physical activity. Previous studies have looked at the return to sport in athletes, in a general population, where rehabilitation is not as controlled as within a captive military population. In this study, a longitudinal prospective epidemiological database was assessed to determine the incidence of stress fractures and the time taken to rehabilitate recruits to preinjury stage of training. Findings demonstrated a background prevalence of 5% stress fractures in Royal Marine training; femoral and tibial stress fractures take 21.1 weeks to return to training with metatarsal stress fractures being the most common injury taking 12.2 weeks. Rehabilitation from stress fractures accounts for 814 weeks of recruit rehabilitation time per annum. Stress fracture incidence is still common in military training; despite this stress fracture recovery times remain constant and represent a significant interruption in training. It takes on average 5 weeks after exercise specific training has restarted to reenter training at a preinjury level, regardless of which bone has a stress fracture. Further research into their prevention, treatment, and rehabilitation is required to help reduce these burdens.

## 1. Introduction

Stress fractures are well recognised in military training and athletes, with the first reported case being identified in 1855 by Breithaupt [1] and the first imaging of a stress fracture recorded by Stechow in 1897 [2].

The incidence of sustained stress fractures in military recruits can be as high as 12% [3], as compared with a rate of 21.1% of elite athletes [4] and 1% of the general population [5].

Trone et al. [6] suggest that recruits who sustain a stress fracture during basic training are over four times more likely to be discharged from training programmes, demonstrating that these injuries can be responsible for a significant portion of attrition in military training with consequent financial implication for military budgets.

Furthermore, after initial rehabilitation, recruits who suffered a stress fracture during basic training are at higher risk of sustaining stress fractures during subsequent training (10.6% incidence within one year of injury, versus 1.7% in injury-free recruits) [7], thereby increasing working days lost to injury and the accompanying financial burden.

Royal Marine training is conducted at the Commando Training Centre in Lympstone, Devon. Between 55 and 60 recruits join at fortnightly intervals throughout the year, excepting leave periods. Spanning 32 weeks, the basic training programme is the longest in the western world, with the average recruit taking a mean time of 37 weeks to complete the syllabus [8]. Training is progressively arduous over this period, culminating in the commando tests, comprising a 30-mile run cross-country, 9-mile run on roads, a 7-mile run cross-country (including through water and tunnels), an aerial assault course, and a week-long final exercise over difficult and varied terrain.

Any recruit who sustains an injury requiring longer than a week to rehabilitate is removed from training and placed in the rehabilitation company. This offers inpatient physiotherapy, remedial physical training, and ongoing military training until the recruit is deemed fit enough to return to mainstream training as a result of passing a predetermined set of “exit” criteria.

Currently, little is known about the length of time required to rehabilitate recruits from stress fractures and return them to their preinjury level of physical activity; previous studies have only looked at the return to sport in athletes, in a general population, where rehabilitation is not as controlled as within a captive military population [9].

We present the largest known study involving stress fractures and their controlled rehabilitation using a longitudinal prospective epidemiological database to determine the incidence of stress fractures and the time taken to rehabilitate patients to their preinjury stage of training.

## 2. Materials and Methods

All patients who were diagnosed with a stress fracture whilst undergoing commando training between April 2004 and April 2008 were treated in the rehabilitation unit with their diagnosis and rehabilitation time prospectively recorded. During this time 4200 Marines started commando training. Patients were diagnosed on plain radiograph taken at initial presentation, repeated at two weeks if symptomatic with an initial negative radiograph. Patients who had a second negative radiograph and were still symptomatic were referred for Magnetic Resonance Imaging to confirm the diagnosis.

Patients were removed from training upon initial presentation and placed into a rehabilitation company, where they initially underwent inpatient based physiotherapy and rehabilitation to recover from their injury and return to training.

The rehabilitation system was divided into two parts, with the first part focusing on the recovery from the initial injury (Group A). When patients were fully recovered from their condition they were moved to a second group (Group B) which focused on regaining fitness and military skills up to the required level for them to return back to training at their preinjury stage.

## 3. Results

During the study period there were 220 stress fractures sustained in 208 patients giving a background prevalence of 5%.

The most common stress fractures are as illustrated in Table 1.

The metatarsals were the most common bone type involved with the third metatarsal being the most likely bone to be fractured. The distribution of metatarsal fractures is as detailed in Table 2.

7.69% of patients with metatarsal stress fractures sustained multiple fractures in the same foot, with one patient having a concurrent stress fracture of his tibia.

81% (17 of 21) of the femoral stress fractures were located in the femoral neck, with the remaining 19% being located in the metaphysis of the femur.

**Figure 1:**
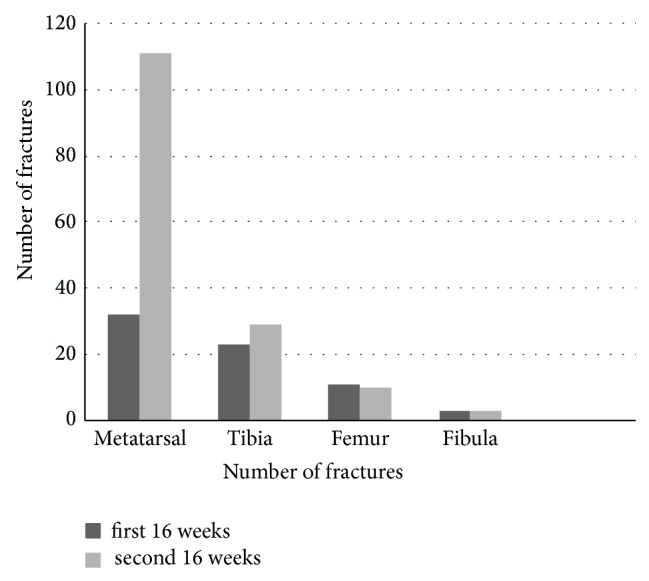
Time of occurrence of stress fracture and anatomical distribution.

Figure 1 demonstrates whether the fractures were sustained in the first part of second part of training, 53% (9/17) of the fractures found in the femoral neck occurred during the second half of the 32-week training program as compared to 78% (111/143) of metatarsal fractures (*P* < 0.0001). Only one metatarsal stress fracture occurred within the first 4 weeks of training.

**Figure 2:**
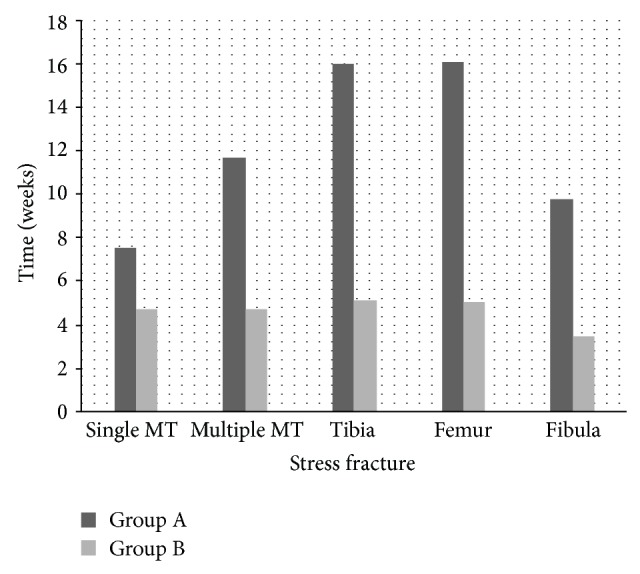
(Group A) Rehabilitation period from injury (Group B). Time in program for regaining fitness and military skills up to allow return to training at their preinjury stage.

Figure 2 demonstrates how long each fracture took to recover in the two stages of rehabilitation. The mean rehabilitation time was as follows: single metatarsal 12.2 (±1.3 95% CI) weeks (range 8–50 weeks); multiple metatarsal fractures 15.4 (±1.2 95% CI) weeks (range 12–18 weeks); tibia fractures 21.1 (±3.4 95% CI) weeks (range 10–47 weeks); fibula fractures 13.3 (±6.5 95% CI) (range 7–23 weeks); and femoral fractures 21.1 (±4.1 95% CI) weeks (range 11–43 weeks).

Stress fractures account for 814 weeks of recruit rehabilitation time per year.

20 (9.6%) of patients had other medical diagnosis at the same time as their stress fractures. The most common diagnosis (25%) was anterior knee pain, with 14/20 (70%) having another lower limb condition, 6/20 (30%) having an infection, 1/20 having a concurrent stress fracture, and 2/20 having an acute trauma fracture.

## 4. Discussion

During the 4-year study period there were 4200 recruits in Royal Marine training sustaining 220 stress fractures at a prevalence of 5%. Similar studies estimate the annual incidence of stress fractures among groups of athletes and military recruits to be between 5 and 30% [10]. Our rate of diagnosed stress fractures is at the lower end of previously reported incidences when compared with similar study groups. With the multifactorial etiology of stress fractures [11] it is difficult to speculate on the precise reasons for the relatively low incidence within our group, although this may reflect a reluctance to present to the medical centre for fear of having to change their training group as a result of injury.

One possible explanation can be derived from a directly comparable study undertaken by Ross and Allsopp in 2002 [12]. This study looked at stress fracture rates in recruits undergoing basic training at the Commando Training Centre both before and after the installation of the Revised Common Recruit Syllabus (RCRS), commissioned to optimise the training programme with respect to reducing injury rates. Results demonstrated a statistically significant reduction in stress fracture rates with the more physiologically progressive RCRS syllabus (3.8%) versus the original training programme (7%), although in their study 30% of patients in the new syllabus were still in training when data collection was completed which may explain our slightly higher incidence of 5%.

Ross and Allsopp argued that RCRS has had a beneficial effect on the incidence of stress fractures; our results support a lower level of stress fractures than prior to the implementation of RCRS despite multiple changes to Royal Marine training since 2002.

Another possible reason for a low incidence of stress fractures is that most fractures were diagnosed by radiograph. It has been recognised that radiographs do not usually contain positive signs associated with stress fractures until two weeks after the onset of symptoms [13]. In this study recruits were diagnosed as having a stress fracture after symptomatic presentation with positive radiograph findings. If symptomatic with a negative initial radiograph, a further radiograph was taken at two weeks. Those who were symptomatic with two negative radiographs proceeded to Magnetic Resonance Imaging (MRI) to confirm the diagnosis. Whilst we have advocated the use of MRI as an initial investigative choice and this remains the UK military policy on suspected stress fractures [2] rapid access to MRI is not always possible, and as a result a pragmatic approach is applied. As a result of this approach, a significant period of time can elapse from presentation of symptoms until radiological diagnosis of stress fracture; however, as all recruits are treated symptomatically in Group A, regardless of diagnosis, a delay in obtaining a definite diagnosis does not cause delay in starting rehabilitation in our study, and we therefore believe that our results demonstrate accurate rehabilitation time for stress fractures.

Giladi et al. [13] advocate a high clinical index of suspicion and early referral for MRI to enable prompt diagnosis among at-risk populations, though definitive evidence is lacking as to whether early diagnostic MRI has significant effect on rehabilitation time from injury and further research into this is necessary.

The pattern of distribution of stress fractures in this study correlates well with previous studies; metatarsals sustain the greatest number of injuries in athletic and military populations [12, 14–17], with fractures of the third metatarsal being the most common although our results would suggest that a higher proportion of stress fractures are 3rd metatarsal (45%) than reported in other studies [12]. Previous research suggests that the third metatarsal is the most common site as a result of increased ligament support of the middle metatarsals resulting in relative resistance to movement, thereby increasing stress forces placed upon this bone [18, 19].

In 2006, Dixon et al. [20] undertook a study on Royal Marine recruits both with and without a history of third metatarsal stress fractures and discovered significant differences in dynamic biomechanical variables of the forefoot, concluding that future successful intervention to reduce the incidence of this injury will likely focus on this area; however, despite these findings, our results show that 3rd metatarsal stress fractures still represent a problem in military training.

Garcia et al. (1987) [21] demonstrated that a majority of metatarsal stress fractures occur in the first 3 weeks; the postulated reason for this is that during the early phase of bone remodeling excessive resorption temporarily decreases the ability for the bone to withstand force [21]. Our results demonstrate that metatarsal stress fractures occur later in training with less than 1% occurring in the first 4 weeks. This may be because Royal Marine training now has a gradual increase in intensity in the first few weeks, in order to allow more time for complete remodeling processes to occur, but in the second half of training there is a sudden increase in mileage as the recruits progress from basic infantry training to more arduous commando training.

Femoral stress fractures are uncommon [22, 23], representing only 10% of the fractures identified in this study. Of these 81% of them involved the neck of femur, with the remaining 19% being located in the metaphysis. A recent study undertaken on recruits based in Northallerton (2008) [22] documents a comparative femoral fracture rate of 8% of all diagnosed stress fractures, whilst Royal Marine training is longer; this does not appear to affect the proportion of femoral stress fractures when compared to other British military training.

Peak presentation time of femoral stress fractures has been debated, having been documented at weeks 13–16 of training [22] and weeks 4–7 of training [23]. Our study demonstrated that 53% of the diagnosed femoral stress fractures occurred during the second half of the training, indicating an equal spread of presentation throughout the 32-week course.

Femoral stress fractures, though less common than fractures at other lower limb locations, represent a more severe complication of training [2, 22, 24]. Unprotected, undisplaced fractures can lead to displaced femoral neck fractures, which carry a 63% complication rate even with optimum treatment [23]. Giladi et al. (1986) [13] showed that 10.6% of recruits who suffer a femoral stress fracture would sustain recurrent stress fractures within one year, demonstrating the gravity of the diagnosis.

The mean rehabilitation time in our study is similar to comparable studies [12]. A mean rehabilitation time for single metatarsal fractures was found to be 12.2 weeks, which increases to 15.4 for multiple stress fractures. There is a significant difference between rehabilitation time for metatarsals and stress fractures in the tibia and femur; however there was no significant difference between stress fractures in the tibia and femur both taking 21.1 weeks. Our results support previous findings with regard to total rehabilitation times [12].

Our results differ from previous studies [12] as we can identify when patients were able to return to activity specific training and also when they returned back to their previous training level. Recruits entered Group B when they were able to restart mainstream training activities and exited Group B back to recruit training after they had passed the appropriate fitness assessment for their level of preinjury training. Apart from fibula fractures where the numbers were quite small, the mean time in Group B ranged between 4.7 and 5.2 weeks, which probably represents the time required to regain cardiovascular and muscle strength fitness and is unrelated to type of stress fracture. Therefore, we believe that future research should be focused on trying to reduce the time required before activity specific training can be commenced.

Our results would suggest that the rehabilitation time required to recover from stress fractures has not decreased since the earlier studies [12] with the mainstay of treatment still being a period of rest from training sufficiently long enough so as to allow healing to occur. There is a requirement to investigate if patients can be physically loaded earlier in the recovery phase and if there are any pharmacological interventions, which can decrease rehabilitation time, in order to reduce the impact of stress fractures on patients sporting and military activities.

It is recognised that the prevention of stress fractures is difficult [13] due to the multifactorial nature of the injury. As such, the fundamental aspects of treatment remain early diagnosis, early identification of symptoms, and a sufficiently long pause in training so as to allow healing to occur [13, 22, 23, 25], Our results would suggest that stress fractures still remain relatively common in military training and that further studies into the prevention and causes of stress fractures are required.

## 5. Conclusion

Mean rehabilitation times vary with the location of the stress fracture. Effective treatment relies on a high index of clinical suspicion, early detection of symptoms, and early diagnosis to reduce rehabilitation time [26].

Despite awareness of the injury and relative lowincidence as compared to similar population groups', stress fractures represent a significant burden in RM training. Stress fractures account for approximately 814 weeks of recruit rehabilitation time per year at an estimated cost of *£*1500 per recruit per week, meaning that total costs exceed 1.2 million pounds [8] per annum warranting further research into their prevention, early detection, and improved recovery times.

## Figures and Tables

**Table 1:** Stress fracture type.

Fracture	Number
Metatarsals	143 (65%)
Tibia	52 (24%)
Femur	21 (10%)
Fibula	6 (3%)

**Table 2:** The distribution of metatarsal fractures.

Metatarsal	Number
1st	0
2nd	38
3rd	99
4th	5
5th	1
